# Immunological considerations in *in utero* hematopoetic stem cell transplantation (IUHCT)

**DOI:** 10.3389/fphar.2014.00282

**Published:** 2015-01-06

**Authors:** Andrea I. Loewendorf, Marie Csete, Alan Flake

**Affiliations:** ^1^Department of Obstetrics and Gynecology, David Geffen School of Medicine, University of California, Los AngelesLos Angeles, CA, USA; ^2^Chief Scientific Officer, The Huntington Medical Research InstitutesPasadena, CA, USA; ^3^The Children's Hospital of Philadelphia, Children's Institute of Surgical SciencePhiladelphia, PA, USA

**Keywords:** *in utero* hematopoetic stem cell transplantation, fetal alloresponse, maternal alloresponse, central tolerance, regulatory T cells (T-Regs)

## Abstract

*In utero* hematopoietic stem cell transplantation (IUHCT) is an attractive approach and a potentially curative surgery for several congenital hematopoietic diseases. In practice, this application has succeeded only in the context of Severe Combined Immunodeficiency Disorders. Here, we review potential immunological hurdles for the long-term establishment of chimerism and discuss relevant models and findings from both postnatal hematopoietic stem cell transplantation and IUHCT.

## Terminology

Central tolerance: An immune mechanism for specificity of the immune response set up in the thymus. Functional central tolerance prevents T cells with a high affinity to “self” from exiting into the periphery.

Peripheral tolerance: Immune protocols that mediate specificity of the immune response, other than central tolerance. All the following can be considered peripheral tolerance:

Dominant tolerance: A tolerance mechanism that can override other tolerance mechanisms such as Tregs (regulatory T cells) or myeloid-derived suppressor cells with capacity to suppress effector mechanisms of other cells.
Regulatory T cells: Specialized CD4+FoxP3+ T cells that inhibit proliferation and effector functions of other immune cells via several mechanisms. Most studied for their effect on CD8+ T cells but potentially also important for regulation of other effector cell types.
Clonal deletion in reference to exhaustion: A type of terminal differentiation in T cells (CD4 and CD8) elicited by continuous presence of antigen. T cells gradually lose functionality in a stepwise, strictly controlled process and sometimes die as a function of continued antigen presence. Partial exhaustion is sometimes reversible.
Anergy: Unresponsiveness of T cells previously stimulated with their cognate antigen in the absence of an appropriate second signal. Anergic T cells do not execute normal effector function.

*In utero* hematopoietic stem cell (HSC) transplantation is not a standard clinical approach, but with greater understanding of the immune system and its development, as well as the disease processes that are optimally treated *in utero*, the procedure may become more widely used. Stem cell transplantation, as any other transplantation is subject to potential rejection reactions by the hosts' immune system that recognizes the tissue antigens of a different genetic makeup as “foreign” and elicits an immune attack. This attack, if not properly controlled by immune-suppressants, can lead to graft damage and ultimately graft loss. Transplantation of hematopoetic stem cells *in utero* seeks to take advantage of the early developmental stages of the fetal immune system and elicit a dampened immune rejection or perhaps achieve full graft tolerance. While attractive in theory, the practical outcomes of *in utero* hematopoetic stem cell transplantation have been disappointing for a multitude of reasons. Here we review the complex issues pertaining the immune system that have bearing on *in utero* hematopoetic stem cell transplantation for a general clinical audience.

## Central and peripheral tolerance

The main function of the immune system is to fight off infection of a huge variety of pathogens. To successfully fight “foreign” invaders without damaging the host, the immune system has to sort through enormous antigenic diversity to recognize the difference between “self” vs. “non-self.” Allogeneic transplants (solid-organ and cell transplants) are not “self” from the recipient perspective and hence are attacked by the recipient immune system (allorejection) necessitating the administration of immunosuppressants. However, successful HSC transplants will actively participate in the immune system as they give rise to all white cells. Additionally, the special situation of HSC transplantation in the context of the fetal immune system during IUHCT warrants specific considerations of the developing recipient environment to ensure graft survival.

Several fundamentally different processes establish necessary self-tolerance and are also involved in (allogeneic) graft-tolerance: central tolerance, exhaustion/peripheral deletion and regulatory T cells (Treg).

Central tolerance results from deletion of self-reactive T cells in the thymus (Rothenberg, 1992). This form of tolerance prevents cells that are stimulated by a self-antigen from migrating into the periphery where they can cause autoimmune damage, and defines the immunological “self,” resulting in a state of “ignorance” to self-antigens. *Ex vivo* stimulation of a central tolerant T cell population with self-antigens does not result in activation or proliferation. Central tolerance is effective but not complete and a few self-reactive T cells can migrate into the periphery despite a functioning central tolerance mechanism (Griesemer et al., 2010). Largely, these self-antigen-specific T cells do not exert autoimmune function in the periphery because they are suppressed by a T cell population which inhibits immune effector functions, the Tregs (Takahashi et al., 1998; Sakaguchi, 2003). A population of cells that is tolerant only because of Tregs *is* stimulated by self-antigen but so are the inhibitory Tregs that prevent self-reactive effector functions. Hence, Tregs establish a state of “active tolerance” toward an antigen. *Ex vivo* stimulation of this cell population with self-antigen also does not result in activation or proliferation. However, when Tregs are depleted in mixed lymphocyte culture systems, stimulation with self-antigen results in activation and proliferation of self-reactive T cells (essentially an “unmasking” of previously inhibited responses). While central tolerance and T regs both mediate non-reactivity to self, the mechanisms by which tolerance is established are very different. There is another distinction between the two mechanisms: Central tolerance is dependent on localization to the thymus while Tregs are mobile, and when transferred to another host, can and will perform appropriate suppressive function if properly stimulated and maintained (Asseman et al., 2000).

Another mechanism that results in a non-reactive T cell pool is T cell exhaustion. T cell exhaustion occurs when T cells are chronically exposed to antigen under inflammatory conditions. Over time, these T cells lose the ability to provide effector functions and die. T cell exhaustion has been observed in persistent infections and has been postulated as a mechanism by which alloreactive T cells decline in functionality and are deleted in solid-organ transplants.

**Figure 1:**
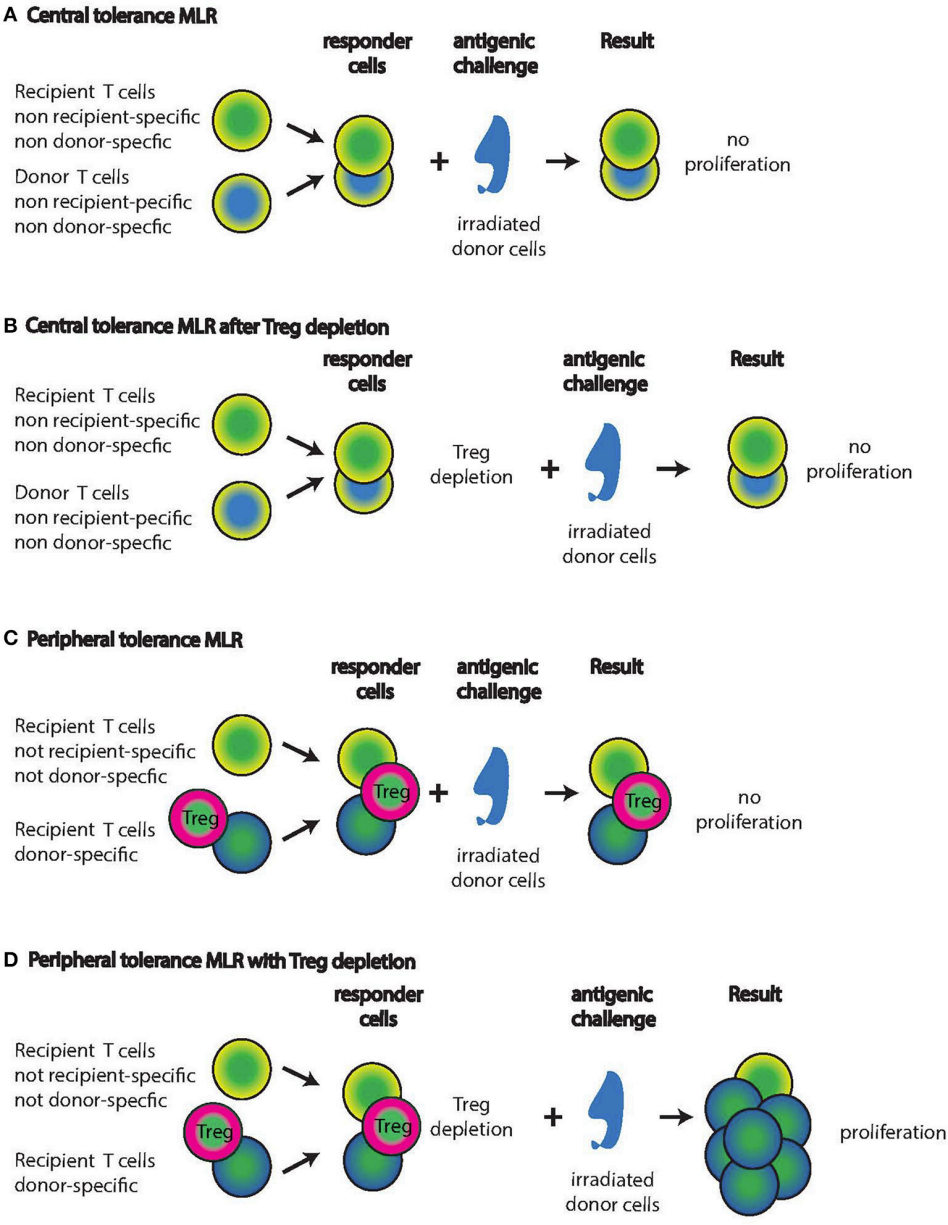
**Mixed Lymphocyte Reactions (MLR) with central tolerant or peripheral tolerant lymphocyte populations from IUHCT graft recipients**. Recipients of IUHCT carry mixed hematopoietic chimerism. **(A,B)** In central tolerance, thymic selection will only allow recipient T cells that are non-recipient and non-donor specific to persist (yellow/green cells). Similarly, donor cells are only allowed to persist if they are non-recipient and non-donor specific (blue/yellow cells). Exposure of these two responder populations to irradiated donor cells will not elicit a proliferative response **(A)** even after the deletion of Tregs **(B)**. **(C,D)** In peripheral tolerance, recipient T cells with specificity for the donor are allowed to progress through thymic selection into the periphery (Green/blue cells) but effector functions and proliferation are inhibited by Tregs (Green/pink cells). Exposure of these three responder populations to donor cells will not result in proliferation **(C)**. After the deletion of Tregs, the recipient T cells with donor specificity will proliferate vigorously in response to donor cells **(D)**. Note: It is unknown whether the Tregs are donor-derived or recipient-derived or both.

In classical *in vitro* mixed lymphocyte reactions (MLR) all the mechanisms discussed above result in non-reactivity of the responder T cells (Figure 1). But that result is misleading in that additional experiments (some of which are technically challenging) can unmask activity of T cells in these “negative” MLR studies. These studies are really controls needed to interpret results of engraftment protocols with confidence, especially since the protocols are often not standardized, and use very different models, time points, cell sources etc. The absence of follow-up studies also highlights the problems in reproducing results or adapting protocols from one model system to another.

## The mechanism of central tolerance

Hematopoietic progenitor cells mature in the thymus, and then are selected to generate a functional, self-tolerant peripheral T cell pool. The outer region (cortex) of the thymus supports the early stages of T cell development, when thymocytes are selected for ability to interact with self-MHC molecules. Cells that survive this positive selection process travel on to the inner thymus (medulla), where negative selection against self-tissue-specific antigen recognition takes place (self -peptide-tolerance).

### Thymic development

Lymphoid progenitor cells migrate during embryonic development in several discrete waves, coordinated with organogenesis (Le Douarin, 1973; Le Douarin and Joterau, 1975). The E11.5 murine embryonic thymus receives a first influx of a small number of lymphoid progenitor cells (Owen and Ritter, 1969; Fontaine-Perus et al., 1981; Liu et al., 2005; Foster et al., 2008). In humans, the thymus appears roughly 8 weeks after conception at which time the first T cell progenitors originating from the fetal liver and later from the bone marrow migrate into and colonize it (Haynes et al., 1989). By week 10, 95% of the human fetal thymus are lymphocytes (Fernandez and de Alarcon, 2013). In mice, a model that is widely applied in studies of immune development, only small numbers of T and B cells can be found at parturition and the division of the spleen into T-cell and B cell zones (red and white pulp) only occurs in the first week after birth (Burns-Naas et al., 2008). In contrast to this, in humans, small numbers of T and B cells can be found at the end of the first trimester while splenic demarcation occurs at the beginning of the third trimester.

**Figure 2:**
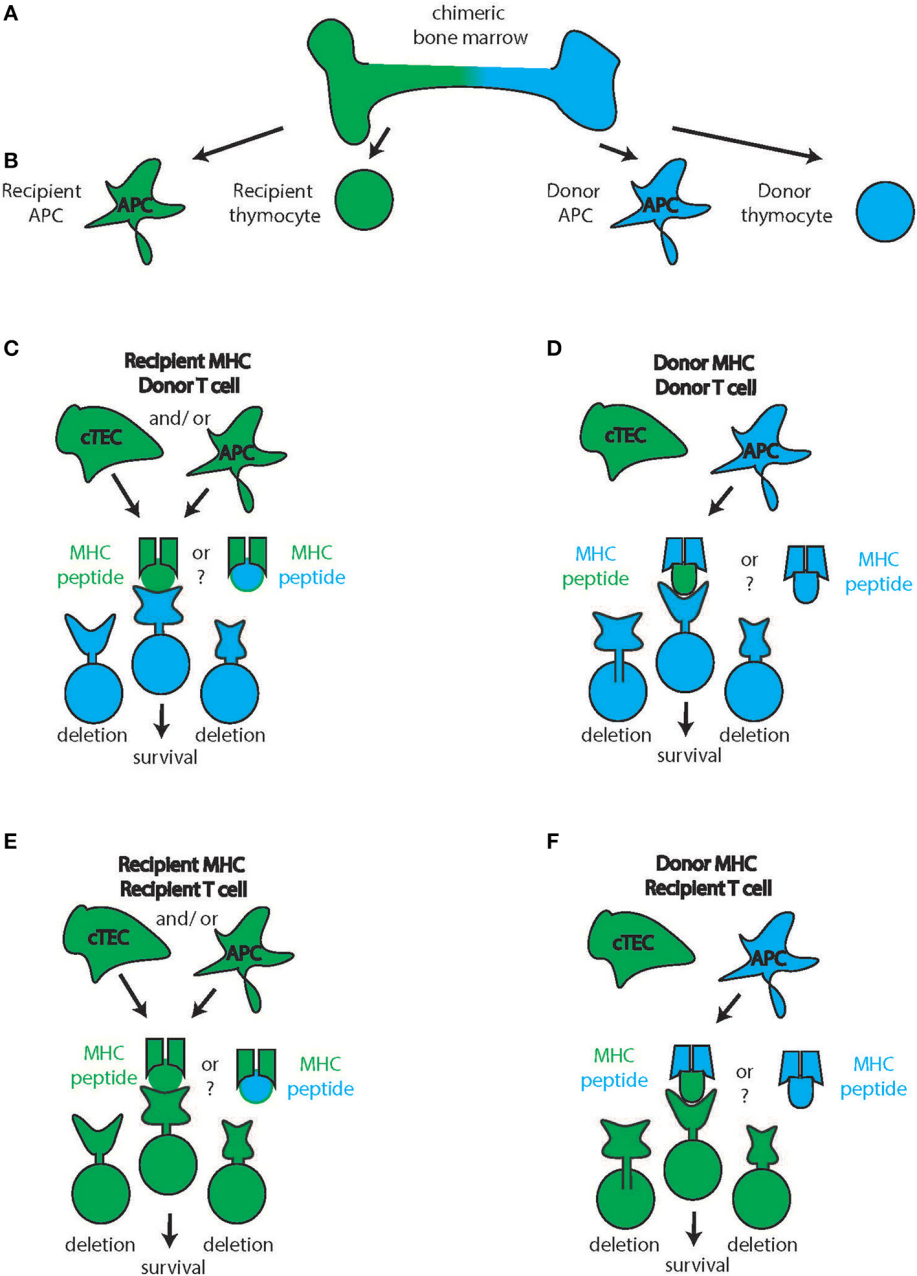
**Cortical thymic T cell selection. (A)** Chimeric bone marrow can give rise to multiple blood products from the two stem cell sources (recipient: green, donor: blue) such as antigen-presenting cells (APC) and thymocytes **(B)** In the thymic cortex, productive interaction with the MHC takes place. Though knowledge is limited, cortical Thymic Epithelial Cells (cTEC) likely will be recipient-derived (**C–F**, green cTEC cell). In contrast, APC are constantly turned over and immigrate from the periphery theoretically allowing for the participation of donor-derived APC in the thymic selection process (**D,F**). Therefore, donor thymocytes can interact with recipient cTEC and recipient APC and hence recipient MHC to receive survival signals **(C)** or with recipient cTEC and donor APC hence interacting with donor APC **(D)** Conversely, recipient thymocytes can interact with recipient cTEC and recipient APC and hence recipient MHC to receive survival signals **(E)** or with recipient cTEC and donor APC hence interacting with donor APC **(D)** The source of the peptides during this process (recipient or donor) is unknown.

**Figure 3:**
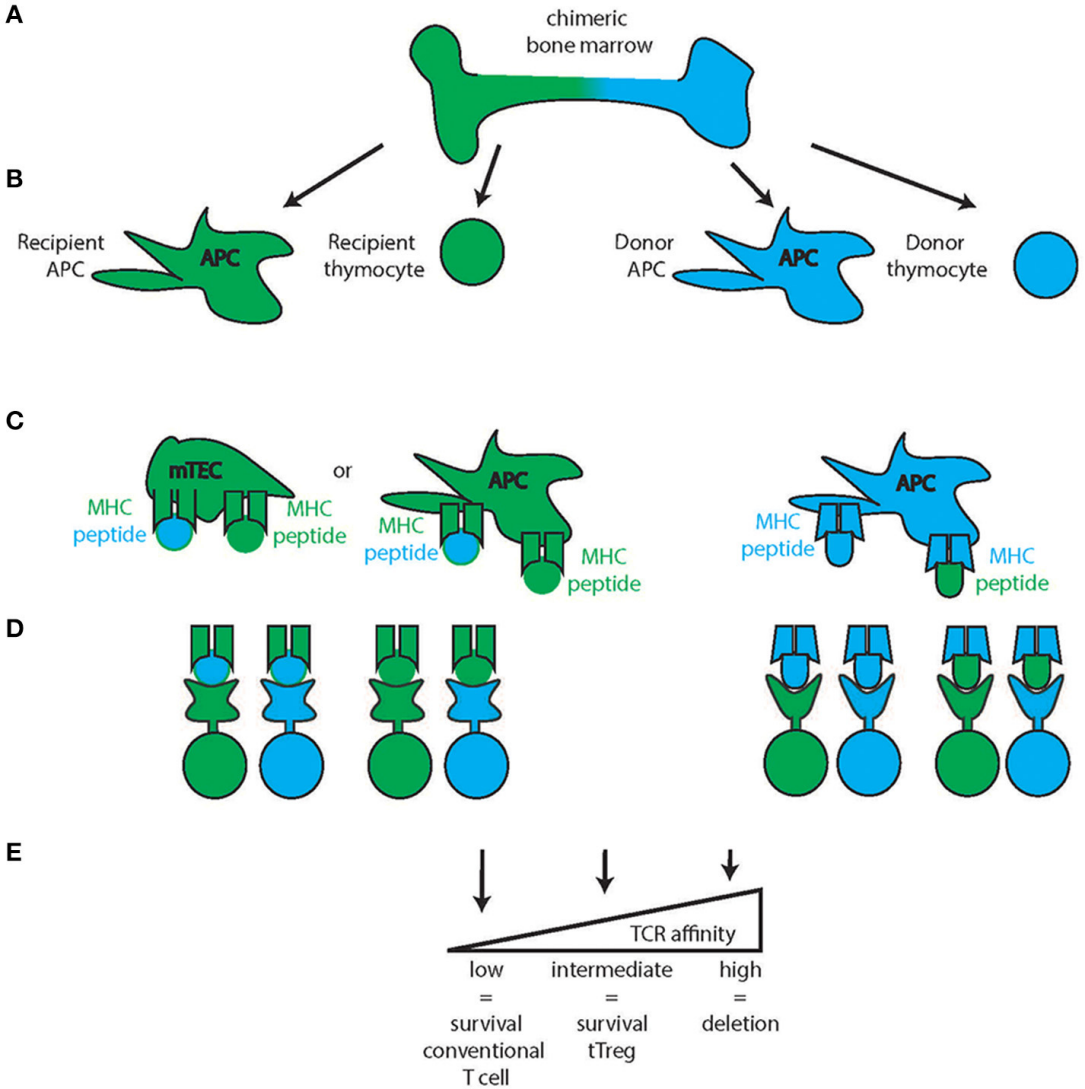
**Thymic medullary selection**. In the second step of thymic selection, T cells are selected for the absence of high affinity self-peptide-self-MHC reactivity. Chimeric bone marrow **(A)** can give rise to multiple blood products from the two stem cell sources (recipient: green, donor: blue) such as antigen-presenting cells (APC) and thymocytes **(B)** While the Medullary Thymic Epithelial Cells (mTEC) are recipient derived (green mTEC, **C)**, medullary APC can be either recipient derived (green, left) or donor derived (blue, right) and all of these cells can either present recipient (green) or donor derived (blue) peptides on their respective MHC. Combined with thymocytes of either recipient or donor origin, several combinations of MHC-peptide-TCR reactivity are possible **(D)** of which those with high affinity will be deleted **(E)** intermediate reactivity will develop into tTregs and low affinity will develop into conventional T cells of the CD4 or CD8 lineage.

The thymus is composed of cells of stromal and hematopoietic origin such as mesenchymal cells, thymic epithelial cells (TECs), endothelial cells, and dendritic cells. Thymic epithelial cells are the main cell type interacting with and providing selective clues to T cells (Figures 2, 3). Based on location in the thymus, TEC are characterized as cortical TEC (cTEC) or medullary TEC (mTEC). Both arise from a common progenitor during thymic organogenesis (Rossi et al., 2006) and, if undisturbed, this progenitor pool continues to support TEC generation and homeostasis in postnatal life (Bleul et al., 2006).

### How do TECs shape the T cell pool?

Initial stages of T cell selection ensure that the resulting pool can successfully “communicate” with cells of the host. To ensure proper communication, T cells must be equipped to engage in productive interactions with proteins that constitute the communication system, the MHC proteins. The cortical thymus selects for this function in a process termed positive selection: T cells with low-affinity interaction of T cell receptors with peptide-MHC complexes receive survival signals while T cells with no or too low affinity engagement die by neglect (Starr et al., 2003). Importantly, T cells with too high an affinity for MHC are also deleted as potentially damaging self-reactive players. Thymocytes travel to the medulla where they interact with mTEC and dendritic cells that present self-antigens under the control of the Autoimmune regulator gene (Aire) (Laan and Peterson, 2013). Thymocytes reactive to presented self-antigens are deleted as potentially self-reactive T cells (also affinity dependent). The T cells reside in this environment for 4–5 days until they are released to the periphery.

### The thymic definition of “self”

The repertoire of self-antigen available for thymic selection is of critical importance as it ultimately defines T cells as self-reactive or not self-reactive. The diversity of this peptide pool is ensured by a remarkable heterogeneity of thymic antigen-presenting cells (APC) and the utilization of unconventional molecular pathways by APC. These pathways ensure unique self-peptide repertoires for both the negative and positive selection steps, with peptides derived from the thymus itself, from the periphery and different developmental stages, including some from stages other than the current developmental state. This remarkably diverse repertoire is ensured by (1) promiscuous gene expression by mTEC, (2) specific antigen processing features including proteasome composition and protease sets, (3) intracellular antigen sampling via autophagy of thymic epithelial cells and (4) extracellular antigen sampling provided by immigrating dendritic cells (DC) that sample the periphery. mTEC express hundreds of self-antigens that are otherwise expressed only in strictly regulated spatial or temporal contexts. In mTEC, proteins are translated for the sole purpose of undergoing degradation, after which they are loaded onto MHC molecules. Regulation of this complex and unique process is still poorly understood but the recent discovery of the transcriptional regulator Aire has shed some light on this process (Anderson et al., 2002). Aire deficiency manifests with autosomal recessive inheritance pattern and causes a polyglandular disorder that classically manifests as spontaneous autoimmunity against the parathyroid and/or adrenal glands, and/or by a mucocutaneous candidiasis infection (termed Autoimmune polyendocrinopathy–candidiasis–ectodermal dystrophy (APECED), also known as autoimmune polyendocrine syndrome–type 1 (APS-1) (Bjorses et al., 1998; Rosatelli et al., 1998; Scott et al., 1998). Aire, amongst other functions, controls the expression of tissue antigens (as detailed above in 1) and regulates intrathymic DC positioning via Aire-dependent XCL1-expression (Lei et al., 2011). In mice, the first Aire + mTEC subsets can be observed at around embryonic day E16 and hence precede the formation of fully mature thymocytes (Zuklys et al., 2000; White et al., 2008). In humans, the kinetics by which APECED- associated autoantibodies appear in pediatric patients suggest onset of Aire-mediated self-tolerance at or before birth (Wolff et al., 2013).

Macro-autophagy enables mTECs to present intracellular cargo on MHC II molecules (Nedjic et al., 2009). Resident thymic DC's are known to sample and cross-present mTEC-derived antigens and blood-borne antigens (Klein et al., 2009). Additionally, about 50% of thymic DC are immigrating DC that already sampled the periphery and imported peripheral peptide cargo. Antigen presentation occurs in two important steps—processing of proteins into MHC-binding peptides and binding of a peptide to an MHC complex. Peptides destined for loading onto MHC class I are generated by the proteasome, a multiprotein complex that executes protein degradation processes dependent on the inflammatory milieu. The housekeeping proteasome generates peptides from proteins under non-inflammatory conditions and the immunoproteasome under inflammatory conditions (Kloetzel and Ossendorp, 2004). Thymic DC and mTECs express a mixture of both types of proteasomes and thus present both partly non-overlapping peptide pools but with careful exclusion of pathogen-associated molecules (Nil et al., 2004; Bonasio et al., 2006; Hadeiba et al., 2012). MHC class II binding peptides are generated in the endosomal/lysosomal compartment by a mixture of proteases including those of the thymus, again mirroring the enormous complexity presented to the developing immune system (Gommeaux et al., 2009).

## Mechanisms of peripheral tolerance

### Regulatory T cells (Tregs)

Thymic selection is a highly efficient process but it is not perfect. It is now accepted that self-reactive T cells escape the deletion process and circulate in the periphery. The reason self-reactive T cells do not cause pathology is that they are suppressed by regulatory T cells (Tregs), a subset of CD4+ T cells defined by intranuclear expression of the transcription factor FoxP3. Thymic Tregs (tTregs) develop during thymic T cell selection from CD4+ clones with moderate-to high-affinity for self-antigens. Peripheral Tregs (pTregs) arise from mature, naïve CD4+ T cells in the periphery when they encounter antigen under certain conditions (Curotto de Lafaille and Lafaille, 2009). Peripheral induction of pTregs is thought to require TCR stimulation of naïve CD4+ T cells in the presence of TGF-β and IL-2 and occurs in the tissues (Chen et al., 2003; Mucida et al., 2007; Curotto de Lafaille and Lafaille, 2009) rather than the circulation. For example, most of the pTregs important for mucosal tolerance to environmental and food antigens are induced in the gut (Faria and Weiner, 2006a,b).

Despite the fact that tTregs essentially follow the same ontogeny as effector T cells, the TCR repertoires of Tregs and conventional T cells are distinct with only limited overlap (Hsieh et al., 2004; Pacholczyk et al., 2006). Although there are still many open questions about tTreg development, an amount of self-reactivity below the threshold for negative selection is likely an important factor driving Treg lineage commitment (Hsieh et al., 2012). The contribution of different populations of APC to tTreg formation was explored in a series of studies utilizing genetically engineered mice. Transfer of MHC II–sufficient or MHC II-deficient bone marrow cells into irradiated RAG-1 deficient mice resulted in comparable frequencies of tTregs generated, suggesting that bone-marrow derived APC are dispensable for tTreg induction (Liston et al., 2008). In turn, experiments in which irradiated mice genetically devoid of co-stimulatory molecules were reconstituted with wild-type bone marrow, tTreg frequency was again unaffected suggesting that bone-marrow derived APCs are capable of tTreg suppor (Proietto et al., 2008; Spence and Green, 2008). Of note, the expression of “normal” TCR diversity and function in tTreg populations generated under these artificial experimental conditions has not yet been fully investigated.

Human fetal tissues in the second trimester display a significantly increased frequency of Treg compared to other developmental stages and locations; the lymph nodes contain about 15–20% of these cells (compared to about 5% in other locales and time points) (Cupedo et al., 2005; Michaelsson et al., 2006). Interestingly, this Treg abundance is not true for the fetal thymus at the same time suggesting that the peripheral fetal Treg either originated from nTreg that expanded in the periphery or that they are iTreg (Burt, 2013). Elegant experiments by Mold et. al. demonstrated that these fetal Treg arise in response to Non-Inherited Maternal Antigens (as further discussed below) and unveiled a general propensity of the fetal immune system to react toward and antigenic challenge with the induction of Treg mediated tolerance (Mold et al., 2008). These observations have led to some speculation about a potential contribution to fetal and neonatal increased sensitivity to infections (increased severity and infections with pathogens that are usually considered commensal flora in adults) and less effective immunization by vaccines (Mold et al., 2008). Importantly, the emerging picture points toward the fact that the human fetal immune system is not inert, non-developed or immature but instead highly active generating tolerogenic responses as further detailed in the model of the layered immune system (Mold et al., 2008).

### T cell exhaustion

T cell exhaustion has been observed in many chronic infections and cancer. It is a state in which T cells exhibit poor effector functions, and express inhibitory molecules and a transcriptome distinct from that of functional effector or memory T cells (Wherry, 2011). Exhaustion is best described for persistent infections with high levels of viral replication such as the human immunodeficiency virus (HIV), or hepatitis C virus (HCV). In those settings, CD8+ T cells lose effector functions in a distinctive hierarchical manner starting with proliferative capacity, IL-2 production, then killing capacity. Severely exhausted (end-stage) cells are unable to degranulate at all and are eventually deleted (Moskophidis et al., 1993; Zajac et al., 1998; Wherry et al., 2003). The precise features of exhaustion seem to vary between infections but in general, low CD4+ helper cells and high viral replication are correlated with greater exhaustion of the CD8+ T cell pool in humans (Wherry and Ahmed, 2004; Blattman et al., 2009; Virgin et al., 2009). In both solid-organ and HSC (including IUHCT) transplantation, the recipient and donor alloantigens are continuously present and hence can potentially induce exhaustion of alloantigen-specific T cells. In fetuses or neonates, exhaustion has thus far not been observed possibly due to the tolerogenic propensity in human fetuses (discussed above).

## What types of tolerance mechanisms have been observed in IUHCT transplantation?

In the case of iatrogenic solid organ allotransplants, tolerance is defined as donor-specific hyporesponsiveness in the absence of pharmacologic immunosuppression (Fung, 1999). Operational tolerance has been reported for different types of transplantation but the underlying mechanisms are not fully understood. In IUHCT and HSC transplantation, graft failure or diminished/disappearing chimerism can be due to two factors or a combination thereof: (1) loss of HSC or their differentiation products due to lack of engraftment or (2) immunorejection of otherwise established, healthy HSC or their differentiated products. Some mechanisms that were elucidated in solid organ or HSC transplantation may apply to IUHCT and will be discussed in this context.

### *In utero* tolerance establishment, the experiments of Billingham, Brent and Medawar

In 1953, Billingham, Brent, and Medawar conducted a series of experiments to test the hypothesis that “mammals and birds never develop, or develop to only a limited degree, the power to react immunologically against foreign homologous (as allografts were known then) tissue cells to which they have been exposed sufficiently early in fetal life” (Billingham et al., 1953). Skin grafts between adult mice of two different strains normally result in rejection after about 11 days. In the 1953 study, mouse pups age E15 or 16 (term is day 21) were inoculated with adult tissue from another strain. Five littermates were born to which skin from the same strain as the *in utero* inoculant was grafted. The grafts were rejected by two, accepted long-term in two and accepted only short-term in one recipient. This experiment demonstrates that transplant rejection can be ameliorated by *in utero* exposure to adult allogeneic tissues. However, grafting these mice tolerant to one strain with skin from yet another strain did result in rejection, demonstrating that the acquired tolerance was indeed specific. Lastly, they used the mice tolerant to the skin graft and transferred cells from a mouse of the same strain that was immunized against the same skin graft into these recipients. They found that these previously tolerant mice now rejected the graft, the tolerance was “broken.” Despite much deeper understanding of immunoregulation today, it is still unclear which tolerance mechanism/s were at work in these experiments by Medawar and colleagues (Figure 1).

### The thymus in IUHCT transplantation

Donor-derived APC transplanted as passenger leukocytes are known to play an important role in early graft recognition and graft loss in solid organ transplantation. This type of antigen presentation is termed direct presentation and will stimulate T cells that can productively interact with the donor-MHC-peptide combinations present on the donor APC. The role of direct presentation diminishes over time as the passenger APC die off. In contrast to solid organ transplants, HSC transplants will continuously generate donor APC over the lifetime of the graft. At later times after solid organ transplantation, rejection is triggered by a process termed indirect presentation and refers to the presentation of donor(allo)-peptides by recipient APC. This type of presentation will be continuously functional throughout the lifetime of the graft, as recipient APC circulate and pick up antigen. First observed in solid-organ transplantation, the kinetics and contribution of direct vs. indirect antigen presentation pathways are much less understood in HSC transplantation and IUHCT.

In animals or humans with mixed bone-marrow chimerism, both donor and recipient HSC can theoretically give rise to the different blood products of all blood lineages. To generate peripheral T cells, donor T cells must be positively and negatively selected in the thymus (Figures 2, 3). The establishment of tTreg-mediated tolerance to the donor can follow different scenarios. As mentioned above, the APC compartment of both the thymic cortex and medulla is in constant turnover with peripheral APC migrating in and new resident APC differentiating locally. If the recipient has hematopoietic chimerism after IUHCT, both APC populations can be either donor-derived or recipient-derived (Figures 2, 3). Additionally, these APC sample blood-borne antigens that can also be donor- or recipient-derived. Under these conditions, the selection of both effector T cells as well as tTregs from both sources of HSC that recognize both the host and the donor as “self” is theoretically possible. This scenario would establish a two-way tolerant state, utilizing mechanisms of both clonal deletion and tTreg generation. Indeed, in a mouse model of IUHCT, both donor (direct presentation) and recipient APC (indirect presentation) were found to participate in antigen presentation and both induced clonal effector cell deletion in the thymus (Nijagal et al., 2013). Interestingly, deletion was also detected in the spleens of animals that did not develop chimerism indicating that transient antigen presence is sufficient for the development of central tolerance.

In a SCID patient with a haploidentical fetal liver and thymus transplant, T cell clones were established after 11 years of healthy life of this patient (no indications of GVHD) (Roncarolo et al., 1988). B lymphocytes were found to be of recipient origin while the T cells were of donor origin. Of the 50 established T cell lines, 15 displayed recipient reactive proliferative and cytotoxic responses (6 CD4+ T cell lines and 9 CD8+ T cell lines) demonstrating that clonal deletion of host-reactive T cells is not complete in this patient. In subsequent SCID patients transplanted with either haploidentical bone marrow or fetal liver stem cells, donor-anti-recipient MLR were non-reactive but again, host-reactive donor T cell clones were isolated (Bacchetta et al., 1993, 1995). However, these effector cells, especially the CD4 T cell clones, displayed reduced cytokine production capabilities and reduced proliferative capacity compared to non-transplanted donor cells. These observations suggest a Treg-mediated suppression of T cell reactivity *in vivo* and in MLR accompanied by an exhaustion mechanism that limits reactivity of the recipient-reactive donor cells that escape thymic selection.

### Regulatory T cells in IUCHT tolerance

In a murine IUHCT model, a higher proportion of Tregs was observed in animals that retained chimerism vs. animals that did not retain chimerism or were not subject to IUHCT (Merianos et al., 2009). The authors speculate that a similar mechanism that leads to fetal Treg induction in response to trafficking maternal cells would be responsible for the observed increase in animals with chimerism. However, in this study, the origin of Tregs (donor or recipient derived) was not further addressed. That question was addressed in a study utilizing breeding schemes of T cell transgenic animals to explore the role of direct vs. indirect presentation after IUHCT (Merianos et al., 2009; Flutter et al., 2010). Similar to the finding of the previous study, both with direct and indirect presentation, the percentage of donor-derived Tregs in the thymus and spleen was significantly increased. However, the absolute numbers of Tregs were not increased but instead the skewed proportions were due to a decrease in effector T cells. While these studies indicate that the donor cells are converted to Tregs and contribute to the tolerogenic environment, whether there is an increase in conversion to Tregs after IUHCT is still somewhat unclear.

### T cell exhaustion/clonal peripheral deletion in IUHCT tolerance

Given that the key factors involved in T cell exhaustion—the continued presence of antigen and inflammation—applies to both solid organ and HSC/IUHCT transplantation, the role of T cell exhaustion needs to be critically evaluated in this context (Valujskikh and Li, 2012).

In IUHCT, the mechanism of T cell exhaustion would be in play if clonal deletion of recipient-reactive donor cells in the thymus is incomplete. In that case, exhaustion of recipient-reactive donor cells would limit GVHD damage that these cells would otherwise cause. Indeed, in SCID patients transplanted with either fetal liver stem cells or haploidentical bone marrow cells, recipient-reactive T cell clones displayed dysfunctional cytokine production demonstrating both incomplete thymic clonal deletion as well as T cell exhaustion of those reactive clones (Bacchetta et al., 1993, 1995). Similarly, in a mouse model of IUHCT, partial clonal deletion and absence of functional rejection of a donor skin graft were observed (Kim et al., 1999). In that model, functionality of the remaining donor-specific T cells could be re-established after the addition of IL-2, indicating that these cells were indeed partially exhausted, albeit this can only be determined with certainty in MLR with and without Treg depletion.

**Figure 4:**
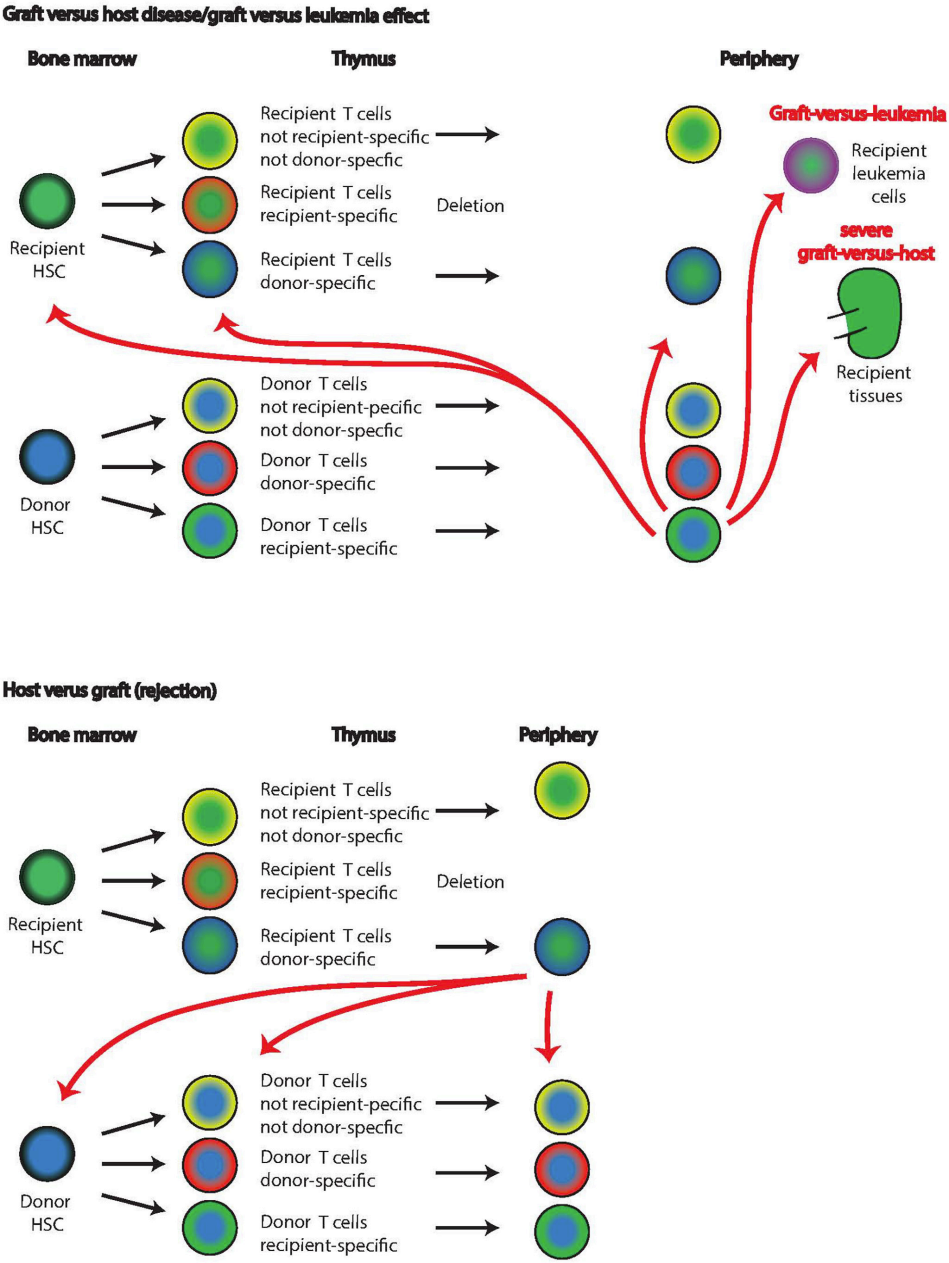
**Graft-vs. host and host-vs. graft disease**. Graft-vs. host disease or graft-vs. leukemia effect is based on the same anti-host reactivity of the graft lymphocytes (top panels). Recipient (green centers) and donor (blue centers) cells undergo thymic selection, and donor T cells with recipient specificity escape into the periphery (lowest row of top panel). These recipient-reactive cells will delete leukemia cells and other host lymphocytes in the periphery. In severe cases, graft-vs. host disease develops and recipient tissues such as the kidney are attacked. The extent of recipient-specific effector cells attacking recipient T cells already in the thymus or HSC in the bone marrow is unknown. Host-vs. graft reactions (lower panels) are the basis of graft rejection in which recipient T cells with donor specificity escape thymic deletion and attack donor cells. While it is known that ultimately disappear from the bone marrow in this case, it is unclear whether this is due to direct deletion by donor-specific recipient cells or another mechanism.

Information about the mechanism of T cell exhaustion can be gleaned from the study of HSC transplantation in the setting of chimerism with non-hematopoietic recipient cells serving as potential APC is similar to IUHCT. Indeed, the antigenic source responsible for donor T cell exhaustion was addressed in a mouse model of allogeneic bone marrow transplantation. Here, a clinical scenario of non-myeloablative transplantation and delayed lymphocyte infusion was modeled (Flutter et al., 2010). Murine recipients were lethally irradiated and reconstituted with mixed bone marrow cells that included T cell-depleted host marrow and T cell-depleted donor marrow with a partial MHC mismatch and a congenic marker allowing identification. After reconstitution and recovery from lymphopenia, donor T cells were infused which led to the elimination of host hematopoietic elements but not to GVHD, modeling a graft-vs.-tumor effect (Figure 4). The donor lymphocytes expanded vigorously up to day 12 and then declined but even after day 60, 5–10% of peak numbers of these alloantigen-primed cells persisted in lymph nodes. However, tests of donor-host cytotoxic activity *in vitro*, showed reduced T cell effector function, proliferative capacity, and IFN-γ production by day 60. This observation of continued presence of donor cells but reduced effector and proliferative capacity is strongly indicative of classical exhaustion. The authors next inquired whether persistent antigen expression by the recipient non-hematopoietic compartment was responsible for donor cell exhaustion. To address this, they performed a similar experiment as discussed above utilizing a transgenic mouse strain that does not express non-hematopoietic tissue antigen (minor HC) genes. In those mice, stimulation of allo-responses occurs only via professional APC while in wild-type mice, both professional APC and non-professional cells such as endothelial cells can present antigen. At day 60, cytotoxicity, recall responses, and memory formation were impaired in wild type mice experimentally. In contrast, in mice devoid of minor HC genes in the non-hematopoietic compartment, none of these features of exhaustion appeared and host-reactive T cells remained fully functional even after 60 days, suggesting that antigen presentation by non-professional APC is responsible for allo-specific T cell exhaustion. These observations strongly indicate that recipient-reactive donor T cells can escape thymic clonal deletion but will acquire an exhausted phenotype in the periphery due to continued presence of “their” antigen in the context of non-professional antigen-presenting cells. The extent to which this is the underlying explanation for T cell exhaustion in the context of IUHCT remains to be determined.

## Immunological barriers to IUHCT

Successful, long-term engraftment of HSC requires successful initial engraftment overcoming the barriers of host-competition, immunological space availability, “receptivity” of appropriate niches to support stem cells and possibly active rejection mechanisms. At later times, graft maintenance and functionality requires both the continuous absence of active rejection mechanisms (or the presence of rejection that is low-level and does not delete the graft) and successful participation of graft-derived cells in the selection and maturation processes to form fully differentiated cells. Failure of any of these requirements may limit engraftment or lead to poor long-term graft function but the participation of each of these components in the final phenotype is sometimes difficult to assess. In IUHCT, the presence of the maternal immune system adds another layer of complexity, the effects of which are only partially understood.

### Facilitating HSC engraftment in IUHCT

Current observations in IUHCT models suggest that successful engraftment starts in the fetal liver from which the cells migrate into the bone marrow. In mice, CXCR4 chemokines follow an SDF-1a chemoattractant gradient for fetal-liver to fetal bone marrow migration. Migrating HSC also express matrix metalloproteinases (MMP-2 and MMP-9) at their leading cellular edge, facilitating degradation of extracellular matrix during migration toward marrow, and they secrete collagen and metalloprotease inhibitors to modify the niche. Hematopoietic stem cells used for IUHCT should express the necessary migratory molecule repertoire and other factors of fetal liver HSC to allow engraftment.

Facilitation of engraftment was further addressed in an IUHCT model in which donor bone marrow was stimulated with vascular endothelial growth factor and stem cell factor (Shaaban et al., 2006). These cytokine-stimulations were chosen to provide a competitive advantage to the graft with regard to homing, establishing residence, proliferation, apoptosis, and bidirectional tolerance. This pre-treatment regimen resulted in higher initial chimerism rates and prolonged survival of skin allografts but long-term engraftment remained unchanged. Expression analysis of relevant homing receptors on the cytokine-treated graft cells did not indicate any changes and host liver homing was not impacted by the treatment. The authors suggest that early progenitor cell subsets did display enhanced homing which could have contributed to the temporary increase in chimerism. They further conclude that cytokine pre-treatment induced cell-cycle activation of normally quiescent progenitors leading to rapid expansion of donor hematopoiesis and increased early chimerism. These observations further underline the importance of long-term monitoring of chimerism in these models and the fundamental disconnect between early engraftment and chimerism and its long-term maintenance. In a series of follow-up experiments, competitive advantage of the graft was achieved by experimental inhibition of CD26 expression in a mouse model of IUHCT (Peranteau et al., 2006). CD26 is a peptidase that cleaves SDF-1a and thereby inhibits its chemoattractant effect on HSC. Blocking of CD26 in IUHCT of bone marrow or enriched HSC resulted in increased homing to the fetal liver, and an increased frequency of animals that developed chimerism with increased levels and prolonged stability.

### Self-antigen and T cell homeostasis in different IUHCT applications

Homeostatic maintenance of T cells requires the interaction with self-MHC loaded with self-antigen and γ chain cytokines. T cells need to interact with self-MHC of the appropriate class loaded with self-peptide to maintain a tonal TCR signal that ensures T cell survival (Jameson, 2002). Under lymphopenic conditions such as in a SCID fetus or after anti-thymocyte treatment, naïve T cells not only survive but proliferate until the “immunologic space” is filled. This process of lymphopenia-induced expansion also necessitates self-MHC self-peptide interactions (Bender et al., 1999; Kieper and Jameson, 1999; Oehen and Brduscha-Riem, 1999). Lymphopenia-induced expansion is limited by the available “immunologic space,” physical space with the appropriate microenvironment and resources such as APCs and cytokines for which T cells compete (Ernst et al., 1999; Surh et al., 2000). Restrictions of the immunological space ensure that the steady-state size of the lymphocyte pool is stable *and* diverse. T cells that expand in response to lymphopenia frequently change phenotype, and acquire the phenotypic and functional properties of memory cells without transitioning through the typical effector intermediates (Cho et al., 2000; Tchao and Turka, 2012). Further, memory autoimmune cells are more resistant to immunodepletion and will proliferate vigorously in response to lymphopenia, resulting in a disproportionate enrichment of memory autoimmune cells (Monti and Piemonti, 2013). Considerably less is known about the antigen sources used in self-peptide/self—MHC presentation during homeostatic T cell interactions than is known about the antigen sources used in thymic selection of T cells. Cell death via apoptosis, an orderly process and an integral part of normal development, does not prompt an immunologic explosion. In contrast, necrosis or the related necroptosis occurs in response to starvation or membrane disruption and, despite production of the same antigens as in apoptosis, does provoke the immune system (Walsh and Bell, 2010; Lu et al., 2014). Thus, the presence of antigen does not absolutely imply its accessibility for a given immunological process such as presentation of self-peptide self-MHC in the steady-state homeostatic context.

### Potential fetal adaptive immune mechanisms of rejection

Current protocols for IUHCT in dog and non-human primate models use T-cell replete bone marrow for transplantation. In the absence of this repletion, engraftment efficiency is greatly reduced (Shields et al., 2003; Petersen et al., 2013; Vrecenak et al., 2014a). These observations suggest that functional T cells are necessary for efficient engraftment, possibly to suppress fetal rejection responses or to create hematopoietic space, but these speculations require further study.

Thymic T cell colonization begins 8–9 weeks after human conception and by 10 weeks, T cells constitute >95% of the cells in the thymus (Haynes et al., 1989). Similarly, B cells start to appear in the omentum and fetal liver at 8 weeks post-conception and in the fetal spleen at ~11 weeks. By week 22, 70% of splenocytes are lymphocytes. NK cells appear in human fetal liver at 5 weeks after conception; at 6 weeks they constitute 5–8% and by 18 weeks 15–25% of fetal liver lymphocytes (Uksila et al., 1983; Thilaganathan et al., 1993). They also constitute 10–15% of cord blood lymphocytes. Alloreactive fetal lymphocytes (lymphocytes that responded to *in vitro* exposure to allogeneic cells by proliferating) are detectable in fetal livers as early as 7.5 weeks of gestation, in thymus at 12.5 weeks, and spleen and blood at 14.5 weeks (Stites et al., 1974). T cell lines generated from fetal livers demonstrate allo-reactivity (Renda et al., 2000a). Though limited numbers of relevant studies have been reported, the complexities of the human fetal immune system are becoming increasingly apparent from comparisons of transcriptional profiles of fetal cells vs. adult cells, revealing fundamental differences (Mold et al., 2010). These studies strongly suggest that phenotypic similarity between fetal and adult immune cells might not extend to functional similarity, and that functionality of fetal immune cells deserves direct investigation. Currently, human fetuses after 14 weeks of gestation are considered immunocompetent with regard to donor-rejection responses (Flake and Zanjani, 1999a,b; Renda et al., 2000b).

**Figure 5:**
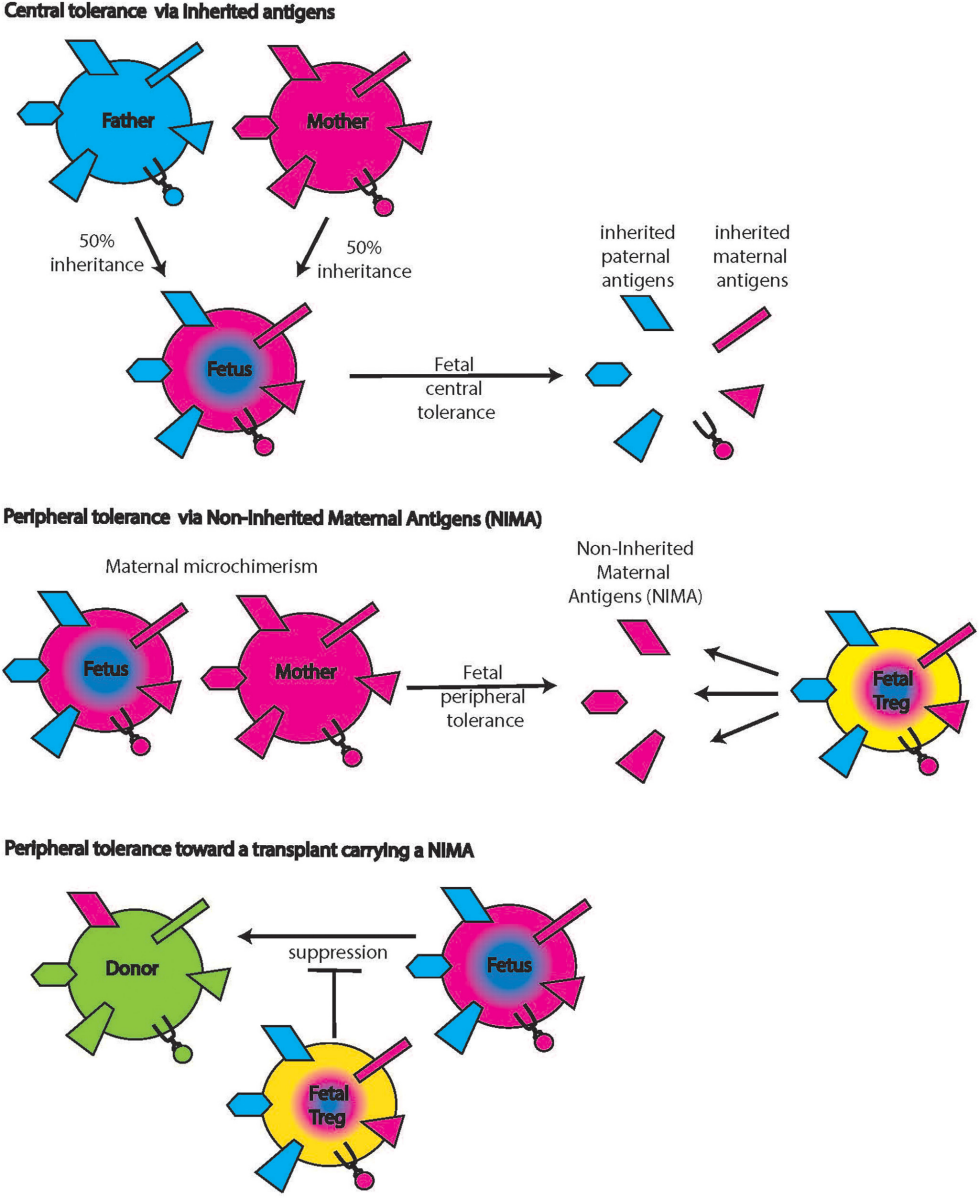
**Non-inherited maternal antigens (NIMAs)**. Central tolerance includes tolerance toward antigens that the fetus inherited from both mother and father (top panels). Maternal cells inhabiting the fetus bring along additional antigens that were not inherited by the fetus (middle panel, left hand side symbols on the maternal cell). The presence of these antigens induces fetal Tregs specific for these NIMAs (middle panels, yellow cell on the right). When donor cells carry NIMAs, these fetal Tregs will suppress allo-reactions (lower panels), an effect that can be either detrimental or desirable.

In an elegant study, Mold et al. demonstrated fetal regulatory T cell responses elicited by non-inherited maternal alloantigens (NIMAs, Figure 5) (Mold et al., 2008). NIMAs are components of the maternal genome, including MHC genes, not inherited by the fetus. Fetal exposure to these antigens occurs via the transplacental migration of maternal cells and can be detected in multiple fetal organs as early as week 14 (Jonsson et al., 2008). The presence of maternal cells raises the possibility of fetal anti-maternal immune rejection and implies parallel development of regulatory elements that limit rejection (reviewed in Burt, 2013). Indeed, Tregs are fairly abundant in the human fetus and the early fetal immune system seems skewed toward tolerogenic responses until effector functions increase and dominate at term (theory of the layered immune system in human fetuses) (Herzenberg et al., 1992). Fetal tolerance of NIMAs was postulated before it was demonstrated experimentally based on epidemiological data showing that maternal donor organs elicit less rejection than paternal donor organs (Burlingham and Benichou, 2012). The conditions under which (semi)allogeneic maternal cells induce fetal Tregs, are also not yet fully understood. The constant, low number of immigrating maternal cells is clearly different from IUHCT in which a large bolus of cells is delivered at once, disrupting the normal cell number ratios and relationships. Better understanding of this mechanism could be useful in the development of protocols to induce Treg tolerance to the IUHCT.

### Rejection by fetal innate immune mechanisms

The role of innate immunity in solid allograft and HSC rejection is poorly understood (Alhajjat et al., 2010; Loewendorf and Csete, 2013). Human umbilical cord blood contains NK cells with mature MHC responsive receptors suggesting that NK self-recognition is functional before birth (Grzywacz et al., 2006; Wang et al., 2007). Insights into the role of innate immunity in IUHCT were gleaned from recent mouse studies in which threshold levels of initial chimerism (>1.8%) were found to predict long-term engraftment and correlated with a donor-tolerant NK response (Durkin et al., 2008). NK depletion in animals with sub-threshold chimerism resulted in abrogation of allograft rejection and the establishment of chimeras. NK cell reactivity depends on a delicate balance of activating and inhibitory receptors and in stable chimeras, the expression of the inhibitory NK receptor Ly49A is down-regulated compared to animals in which chimerism was lost. Initially, Ly49A expression was reduced in all recipients of IUHCT albeit still higher in the animals that subsequently lost chimerism indicating that those animals experienced an inadequate degree of receptor-ligand interaction resulting in failed host NK-cell education during the time of receptor acquisition. In a subsequent study, the transfer of donor MHC to host NK cells (trogocytosis) was shown to correlate with NK cell tolerance (Alhajjat et al., 2013).

### Microchimerism vs. macrochimerism and its role in transplant tolerance and IUHCT

Since the discovery of donor microchimerism in recipients of solid-organ transplants by Starzl et al. donor leukocytes that remain functional through the organ preservation process and enter the host bloodstream were suspected mediators of transplant tolerance (Starzl et al., 1992; Hisanaga et al., 1995; Hundrieser et al., 1995; Schlitt et al., 1995). Chimerism that is not detectable by FACS but only with sensitive PCR methods is generally referred to as microchimerism while macrochimerism is chimerism at levels readily detectable by FACS. Donor leukocytes can persist in the recipient for many years and result in allogeneic microchimerism not unlike maternal-fetal microchimerism (Verdonk et al., 2011). Stable levels of donor microchimerism have been suggested as a marker of transplant tolerance allowing reduction of immunosuppressive drug doses (Ayala et al., 2009). One percentage microchimerism can be sufficient for transplant tolerance in xenogenic animal models (Ildstad and Sachs, 1984). These studies have prompted exploration of chimerism as a tolerogenic mechanism, clinically accomplished by transplantation of allogeneic bone marrow after appropriate host conditioning (Trivedi et al., 2005). Nonetheless, the mechanism of tolerance induced by microchimerism is not completely clear. Early interpretations such as the theory of reciprocal deletion and exhaustion of host and donor effector T cells were based on the absence of donor-reactivity in *in vitro* MLR or killing assays without taking the role of Tregs into account (Starzl et al., 1994). Clinically, various types of chimerism levels from none to high or temporary have been observed without clear correlation to clinical rejection or operational tolerance (Billingham et al., 2003).

In a mouse model of IUHCT, congenic adult bone marrow was used for IUHCT followed by booster injections on days 2, 4, and 7 of life (Milner et al., 1999). The congenic setting ensured that immunologic effects would not confound the observations. Postnatally boosted animals had significantly higher levels of donor cell engraftment as determined in the peripheral blood 6 weeks after birth and the increased levels were stable until the last time point analyzed, 6 months of age. In a subsequent study a booster transfer of allogeneic donor bone marrow was applied after non-myeloablative total body irradiation (Peranteau et al., 2002). In that study, low-level chimerism after IUHCT was enhanced to high-level chimerism depending on the irradiation dose; the mechanism was found to be donor tolerance by the host and a transient competitive advantage of the non-irradiated donor stem cells. Lastly, no evidence of GVHD was observed in these experiments, suggesting that this protocol of prenatal tolerance induction in preparation for subsequent postnatal treatments is a potential clinical strategy. Booster regimens were also found to be possible in canine models, in 2 of the 5 boosted animals the degree of chimerism increased from <1% after IUHCT to 35–45% after postnatal boost with busulfan conditioning (Peranteau et al., 2009). In contrast, postnatal HSC transplantation did not result in detectable chimerism in naïve controls, demonstrating that IUHCT conditioning is crucial to facilitate postnatal minimal conditioning HSCT. Importantly, the phenotype of canine leukocyte adhesion deficiency (CLAD) was ameliorated by the IUHCT serving as a proof of principle that this lethal phenotype can be rescued by IUHCT. The reason for the heterogeneous chimerism levels within one group of experimental animals is unclear. Possibly, a certain threshold microchimerism needs to be established via IUHCT to achieve central tolerance which subsequently allows for the effective postnatal boost but further studies are required to understand this phenomenon.

A study refining the delivery method of IUHCT in the haploidentical canine model recently achieved, for the first time, long-term stable therapeutic levels of chimerism in a large animal model without prior conditioning or GVHD (Vrecenak et al., 2014b). Intraperitoneal injection was compared to intracardiac delivery and the latter achieved far higher intravascular levels of circulating donor cells, higher levels of initial fetal liver engraftment, and subsequent higher long term donor chimerism. Donor-derived renal grafts were transplanted and monitored for rejection to assess the presence of donor specific chimerism vs. tolerance. All recipients with chimerism above 10% showed no acute or chronic rejection at any point and, importantly, no signs of GVHD were observed, further underlining that fetal recipients are less prone to the development of this serious complication. Taken together, these experiments provide important experimental justification to move forward with clinical trials of IUHCT for inherited hematologic diseases.

### Rejection by maternal mechanisms

#### Maternal antibodies in utero and in breast milk

Maternal immunoglobulin G (IgG) antibodies are transported to the placenta via an FcR-mediated process (Malek et al., 1996). The amount of maternal antibody present in the fetal bloodstream increases exponentially during gestation, reaching 50% of maternal blood levels at weeks 28–33, then continues to increase, resulting in higher amounts of IgG in fetal than maternal serum at term (Malek, 2003; Esposito et al., 2012). This maternal antibody protects the baby in the first months of life, and maternal vaccination during pregnancy with the goal of protecting the infant by transplacental IgG transfer has been demonstrated in clinical trials (Esposito et al., 2012). After birth, maternal antibodies are continuously transferred via breast milk and thus provide continuous protection. Potential danger posed to fetuses by maternal antibodies first became evident when fetal hemolytic anemia, resulting from maternal anti-Rhesus antibodies was discovered (Chown, 1954).

In IUHCT, maternal anti-donor antibodies have been shown to play an important role in long-term chimerism maintenance: Merianos et al. demonstrated that maternal sensitization during IUHCT and subsequent transfer of alloantibodies via maternal breast milk induces an adaptive immune response in mouse pups which leads to graft rejection (Merianos et al., 2009). This rejection was not observed if the pups were fostered by dams not subjected to the IUHCT procedure and thus did not produce alloantibodies. In contrast to traditional views that suggest maternal antibodies act by directly binding the target antigen, the authors convincingly demonstrate the elicitation of a fetal effector response, similar to observations by others in the setting of fetal antibody exposure (Greeley et al., 2002; Setiady et al., 2003). The maternal alloantibodies produced in this setting were both IgM and IgG (mostly IgG2a) classes. Although the specific mechanism by which maternal alloantibodies elicit an immune response in the fetus is not yet clear, these observations suggests that maternal sensitization during IUHCT in humans could pose an additional hurdle as the gestation time in humans is much longer, potentially allowing for transplacental IgG alloantibody transport that cannot be avoided (in contrast to breast milk-related alloantibody exposure).

#### Maternal microchimerism

Many maternal cell types contribute to microchimerism of the fetus; cells of myeloid and lymphoid lineages have been identified as well as hematopoietic progenitors (Jonsson et al., 2008). Though maternal microchimerism represents a low percentage of total fetal immune cells, it is potentially relevant for IUHCT. In cord blood, 0.1–0.5% of total T cells are maternal (Hall et al., 1995; Mold et al., 2008). These maternal cells are enriched for cells specific for antigens inherited by the fetus from the father, and can enhance graft-vs.-leukemia effects if the cord-blood recipient shares them (Burlingham and Benichou, 2012). In turn, these maternal cells can elicit rejection responses if the HSC graft carries paternal antigen or other antigens to which maternal cells have been sensitized (Figure 1). To avoid graft rejection caused by maternal chimeric cells, a maternal transplant source or avoidance of paternal inherited antigens is advantageous. MLR detection of preexisting maternal reactivity to a potential HSC donor can help identify such potential rejection responses.

Maternal cells with anti-donor specificity can also directly inhibit the establishment of chimerism in IUHCT (Nijagal et al., 2011). In these experiments, T or B cell deficient dams were bred to wild-type fathers and the fetuses were subjected to IUHCT. Engraftment was significantly improved in dams lacking T cells but not B cells. This improvement was not observed if the graft matched the MHC of the mother suggesting that for initial engraftment, transplacental trafficking of maternal allospecific T cells poses a substantial barrier. Whether these maternal cells directly kill donor cells or whether the loss of chimerism is due to other mechanisms is currently unclear. Additionally, the authors demonstrated a marked increase in maternal T cell and also B cell trafficking to the fetus after IUHCT of allogeneic cells (compared to control pups receiving PBS). Potentially, the presence of the additional antigenic load from the donor cells or the induction of local inflammatory responses to cellular debris or transferred apoptotic cells that confer danger signals attracts more maternal cells.

Altered maternal fetal cellular trafficking in humans has been reported after fetal surgeries, preeclampsia and pregnancy termination but potential roles for these insults in the limitation of chimerism has not been determined (Jeanty et al., 2014).

## Conclusion

The fetal immune system is a complex system that changes dramatically as a function of developmental stage, and to patterns of exposure to alloantigens. Given that direct query of the immune response after IUHCT is difficult, well-designed *in vitro* and *in vivo* experiments are needed to dissect the mechanisms of tolerance and rejection for this potentially important clinical procedure.

### Conflict of interest statement

The authors declare that the research was conducted in the absence of any commercial or financial relationships that could be construed as a potential conflict of interest.
